# How Do Student Prior Achievement and Homework Behaviors Relate to Perceived Parental Involvement in Homework?

**DOI:** 10.3389/fpsyg.2017.01217

**Published:** 2017-07-27

**Authors:** José C. Núñez, Joyce L. Epstein, Natalia Suárez, Pedro Rosário, Guillermo Vallejo, Antonio Valle

**Affiliations:** ^1^Department of Psychology, University of Oviedo Oviedo, Spain; ^2^Center on School, Family and Community Partnerships, Johns Hopkins University, Baltimore MD, United States; ^3^Departamento de Psicologia Aplicada, Universidade do Minho Braga, Portugal; ^4^Department of Developmental and Educational Psychology, University of A Coruña Corunna, Spain

**Keywords:** students’ perceptions of parental involvement in homework, students’ homework time management, time spent on homework, amount of homework completed, prior academic achievement

## Abstract

This study investigated how students’ prior achievement is related to their homework behaviors (i.e., time spent on homework, homework time management, and amount of homework), and to their perceptions of parental involvement in homework (i.e., parental control and parental support). A total of 1250 secondary students from 7 to 10th grade participated in the study. Structural equation models were fitted to the data, compared, and a partial mediation model was chosen. The results indicated that students’ prior academic performance was significantly associated with both of the students’ homework variables, with direct and indirect results linking achievement and homework behaviors with perceived parental control and support behaviors about homework. Low-achieving students, in particular, perceived more parental control of homework in the secondary grades. These results, together with those of previous research, suggest a recursive relationship between secondary school students’ achievement and their perceptions of parental involvement in homework, which represents the process of student learning and family engagement over time. Study limitations and educational implications are discussed.

## Introduction

Homework was defined by Cooper (1989) some years ago as the tasks assigned by teachers to students to be completed outside the class. Epstein and van Voorhis (2012) identified homework as a natural connector of school and home. In these ways, homework is one of the most common school activities involving teachers, students, and parents (Rosário et al., 2015). Recently, however, there have been serious debates in Spanish schools and in other countries about whether or not teachers should assign homework. The debates involve students’ complaints about the time required to do their homework, parents’ complaints about the quantity of homework assigned and their lack of information on how to guide their child on homework tasks, and, teachers’ complaints about the lack of time to design effective homework assignments and deliver feedback to students, and the lack of parental support for students to do their work (Cooper et al., 2006).

There are several connections of students’ homework, parental involvement, and student achievement that must be understood to address questions about the value of homework and improving the homework process. These relationships have been frequently studied across the decades, with most studies confirming a positive impact of homework on student achievement (Rosário et al., 2009; Bembenutty and White, 2013). However, findings vary depending on the research design (Cooper et al., 2006; Patall et al., 2008), nature of measures (i.e., global vs. specific) (Trautwein et al., 2009), students’ grade level (Núñez et al., 2015), and focus of the analysis (e.g., student variables, instructional process variables, or parental involvement) (Núñez et al., 2014). Other studies explored the influence of parental involvement on students’ homework behaviors and resulting achievement (Cooper et al., 2001, 2006; Patall et al., 2008; van Voorhis, 2011; Bardou et al., 2012; Dumont et al., 2012; Kim and Fong, 2013).

A substantial number of studies analyzed the association of different student homework behaviors with students’ academic achievement (Xu, 2010; Núñez et al., 2013b; Xu et al., 2014). However, few studies have explored whether and how students’ achievement levels affect their homework behaviors. This study aims to increase understanding on how students’ levels of achievement are related to their homework behaviors (i.e., homework time spent, homework time management, and amount of homework completed), and how students with different achievement levels perceive the involvement of their parents in the homework process (i.e., control and support).

### Why Are Parents Involved in Their Children’s Homework?

Relationships between parental involvement in homework and academic achievement have been deeply debated and frequently investigated, with inconsistent results (Gonida and Vauras, 2014). Some studies found a positive relationship (Cooper et al., 2001; Pomerantz and Eaton, 2001), others reported a negative relationship between the two variables (Schultz, 1999). Dumont et al. (2012) found both positive and negative relationships, depending on the nature or quality of the involvement. For example, whereas perceived parent–child conflicts about homework were negatively associated with educational outcomes, perceived parental competence and support for students’ self-direction were positively related to achievement. Similar results were obtained by Karbach et al. (2013), who found that academic achievement was significantly and negatively associated with parental control and strict structure (i.e., excessive control and pressure on children to complete assignments, consistent guidelines and rules about homework and school work).

In a recent study, Núñez et al. (2015) found that students’ perceptions of strong control by parents in the homework process was directly and negatively related to academic achievement. The higher the perceived parental homework control, the lower the students’ academic achievement. In the same study, perceived parental homework support was positively related to the achievement of junior high and high school students, but not to that of elementary school students.

Why do parents become involved in children’s homework? The literature suggests several reasons for parents’ involvement: their own motivation (Hoover-Dempsey et al., 1995; Katz et al., 2011); their socioeconomic status (Davis-Kean, 2005); teacher outreach and homework design that encourages engagement (Hoover-Dempsey and Sandler, 1997; Epstein and Van Voorhis, 2001); and their children’s academic functioning (Pomerantz and Eaton, 2001; Grolnick et al., 2002; Cunha et al., 2015), with academic functioning one of the strongest instigators of parents’ attention to homework.

That is, parents are more likely to be involved when children are not doing well in school (Levin et al., 1997; Pomerantz and Eaton, 2001; Ng et al., 2004; Silinskas et al., 2010). In that situation, parents are more prone to display controlling forms of involvement (Pomerantz and Eaton, 2001; Grolnick et al., 2002; Ng et al., 2004; Niggli et al., 2007). Thus, although a major assumption in previous studies has been that different types of parental involvement in homework are related to different levels of school achievement, it is also likely that children’s academic achievement predicts or motivates parents to become involved in homework in particular ways.

### Purpose of This Study

Some studies found that parents’ participation in their children’s academic life (e.g., monitoring progress through conversations with teachers, attending to subjects their children are struggling with) is related to students’ homework completion (Pomerantz et al., 2007) and academic achievement (Wilder, 2013). However, investigations of parental involvement in homework is inconclusive (Patall et al., 2008; Wilder, 2013). Although some authors defend parents’ involvement as a positive practice that can enhance children’s academic success, others describe this support as a time-consuming exercise that frequently generates discomfort, anxiety and conflict in the family (Cooper et al., 2001; Pomerantz et al., 2005a; Patall et al., 2008). However, the majority of findings confirm a positive association between children’s academic functioning (i.e., student achievement and productive homework variables) and parents’ involvement in homework.

Most research has focused on how the context (e.g., family or school) or homework variables (e.g., quantity and quality of homework assignments, parental involvement, students’ homework behaviors) influences student achievement (Trautwein et al., 2002; Hill et al., 2004; Cooper et al., 2006; Pomerantz et al., 2007; Trautwein, 2007; Trautwein et al., 2009; Zhu and Leung, 2012; Karbach et al., 2013; Núñez et al., 2013b, 2015). Few studies, however, *flipped the coin* to examine the inverse relationship. As Nurmi and Silinskas (2014, p. 455) point out, there is a need to analyze findings from a ‘child-directed development’ perspective, in their own words, “to see that children are not only the passive targets of their parents’ behavior, guidance, and parenting practices but they also influence their parents in many ways.” For example, Chen and Stevenson (1989) and Levin et al. (1997) concluded that when children showed low academic skills, their parents were more likely to monitor the amount and quality of their homework. More recently, Silinskas et al. (2010, 2013) analyzed the behavior of first and second grade students. They reported that the lower the children’s literacy and numeracy, the higher the levels of homework help and monitoring displayed by their parents. Silinskas et al. (2013) reinforced these findings, reporting that children’s achievement had an “evocative impact” on their parents’ behavior. Dumont et al. (2013) analyzed the relationship between fifth and seventh graders’ functioning on homework and the quality of their parents’ homework involvement (conceptualized as a multidimensional construct). They concluded that students’ skills (e.g., levels of reading achievement, reading effort, and homework procrastination) predicted the quality of parental involvement in homework (parental control, parental responsiveness, and parental structure).

This study addresses how children’s levels of prior academic achievement affect their perceptions of whether and how their parents are involved in homework. As in previous studies (Núñez et al., 2015), the dimensions of parental involvement in homework are control (i.e., parents’ pressure on children to complete assignments) and support (i.e., the value students’ place on parents’ assistance and the spirit of parents’ help to support students’ self-direction or autonomy on homework). We explore whether and how student achievement and homework behaviors (i.e., time spent on homework, quality of homework management, and quantity of homework completed) promote specific kinds of parental involvement in homework.

Recent studies (Dumont et al., 2013; Silinskas et al., 2013) using longitudinal designs analyzed the effects of children’s achievement on subsequent parental involvement. In both studies, the *direct* relationship between the two constructs was estimated with similar results. The associations were negative, indicating that the lower the children’s achievement, the greater the involvement of their parents. However, Dumont et al. (2013) found the significant negative connection only for low achievement on greater parental *control*, but no significant connection with parental *support*. By contrast, Silinskas et al. (2013) reported a significant negative effect of children’s reading achievement on both parental monitoring (similar to the Dumont et al., 2013 study) and an even greater or stronger negative effect of achievement on parental support (measured as “parental help”) which (Dumont et al., 2013) did not find in their study. The different findings by Dumont et al. (2013) and Silinskas et al. (2013) may be due to the different ages of the participating students (grades 1 and 2 vs. grades 5 and 7, respectively).

Taken together, the data from these studies indicate that in the early elementary grades, students with low achievement prompted parents’ control and support behaviors, whereas at the junior high school level, students’ low achievement prompted significantly greater control by the parents who were involved. In order to extend analyses on how the level of students’ prior achievement affects their parents’ involvement, this study included measures of the students’ homework behaviors as potential mediating variables as described by Dumont et al. (2013) and Silinskas et al. (2013). Prior studies were not conducted with students or parents at the high school level.

**FIGURE 1:**
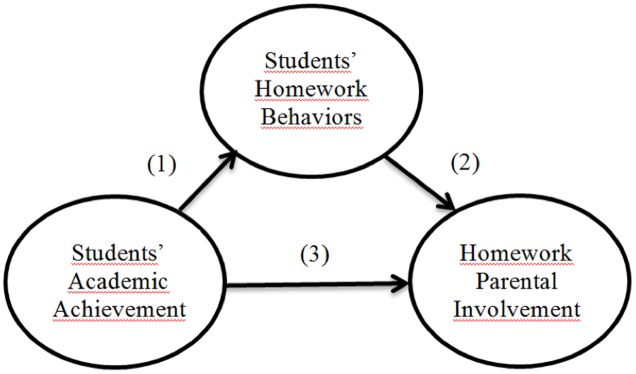
Children’s academic functioning and parental involvement relationship.

For this study of middle and high school students, a structural equation model (SEM) for homework was elaborated and fitted with the following hypotheses (see **Figure 1**):

(1) Prior academic achievement is positively and significantly associated with children’s homework behaviors (i.e., time spent on homework, homework time management, and amount of homework completed);
(2) Children’s homework behaviors are associated with their perceptions of parental involvement in homework;
(3) Prior academic achievement is associated directly and negatively with students’ reports of parental involvement in homework (control and support); and
(4) Perceived parental control and perceived parental support are significantly related.

Previous studies identified grade level as a relevant variable when analyzing the relationships between of academic achievement, students’ homework behaviors and parental homework involvement (e.g., Patall et al., 2008; Skaliotis, 2009; Gonida and Cortina, 2014; Núñez et al., 2015). Thus, in this study the sample was divided into two subgroups (7th and 8th = grades—middle school and 9th and 10th grades—high school) to test the model invariance.

## Materials and Methods

### Participants

A total of 1250 Spanish students from 7th to 10th grade with ages ranging from 12 to 16 years participated in this study. These students attended 68 classes in four urban public schools selected at random from all public schools in Asturias. There were 370 students in grade 7, 346 in grade 8, 257 in grade 9, and 277 in grade 10. Fifty one percent of the participants were male. In the Spanish educational system, compulsory secondary education extends through 9th grade. On average, the families of these students were in the middle class, evidenced by the low percentage of students receiving free or reduced-price lunch (18.7%) as reported in schools’ office data.

### Variables and Measures

Students’ perceptions of parental involvement in homework and students’ reports of their own homework behaviors were gathered in questionnaires administered during one regular class period for about 25 min. Students’ prior academic achievement data (report card grades) was provided by the secretary of each school.

Secondary students in middle and high schools are the main actors in their own education, thus students’ reports about their homework behavior and their perceptions of parental involvement provide important views of the homework process. Teachers’ and parents’ actions and messages must be accepted, understood, and processed by the students, themselves, to motivate learning and promote achievement in school (Bempechat, 2004; Epstein, 2011). It is likely, as this study hypothesizes, that the characteristics of students affect how their parents react to them. The data from students provide a good starting place for understanding the research questions in this study.

#### Parental Involvement in Homework

Two dimensions of parental involvement in homework were assessed: students’ perceptions of *control* exercised by parents and students’ perceptions of *support* provided by their parents. The items were adapted from prior studies (e.g., Carter and Wojtkiewicz, 2000; Trautwein and Lüdtke, 2009; Dumont et al., 2012).

*Students’ perceptions of parental control* were assessed with five items (α = 0.82) (e.g., “My parents are fully aware of me completing all my tasks.”) on a Likert scale with five responses ranging from 1 (completely false) to 5 (completely true). The five items were used to create a latent variable (Parental Control) for the SEM analysis.

S*tudents’ perception of parental support* was computed from student responses to three items (α = 0.80) (e.g., “When I have to do homework, explanations by my parents are very useful.”) using the same scoring system as for parental control. A latent variable (Parental Support) was built from the three items for the SEM.

#### Student Homework Behaviors

Variables of homework behaviors were selected from a pool of items used in other studies (e.g., Núñez et al., 2013b, 2015) to create the latent variables.

*Time spent on homework* was calculated from student responses to two items (α = 0.70): “How much time do you usually spend on homework each day, Monday through Friday?” and “How much time do you usually spend doing homework during the weekend?” The items were scored on a five point Likert scale, ranging from 1 (*less than 30 min*), 2 (*30 min to 1 h*), 3 (*1 h to hour and a half*), 4 (*1 h and a half to 2 h*), to 5 (*more than 2 h*.

*Homework time management* was calculated from student responses to two items (α = 0.72): “When I’m doing my homework, I get distracted by anything that is around me,” and “When I start homework, I concentrate and do not think about anything else until I finish (reverse coded).” These items were rated on a five-point Likert scale, ranging from 1 (*always*) to 5 (*never*).

*Amount of homework completed* was assessed from student responses to the following question: “Usually, how many tasks do you complete from the assigned homework?” This item was rated on a five-point Likert scale, ranging from 1 (*none*) to 5 (*all*).

#### Prior Academic Achievement

Prior academic achievement was obtained from students’ report card grades in mathematics, Spanish language, English language, and social sciences at the end of the academic year (June) (see Núñez et al., 2015). The grades for the four subjects were used to build a latent variable (Prior Academic Achievement). The measurement scale of this variable ranged from 0 to 10 with 5 as a passing grade.

### Procedure

Participating students were volunteers with approval from their parents. Researchers signed agreements with the collaborating school boards to conduct workshops for participating teachers and for parents on the results and educational implications of the research. All measures except prior academic achievement were collected in October at the beginning of the school year. In the current study the measure for prior academic achievement refers to students’ achievement at the end of the previous school year and is used as an explanatory variable.

### Data Analysis Strategy

**FIGURE 2:**
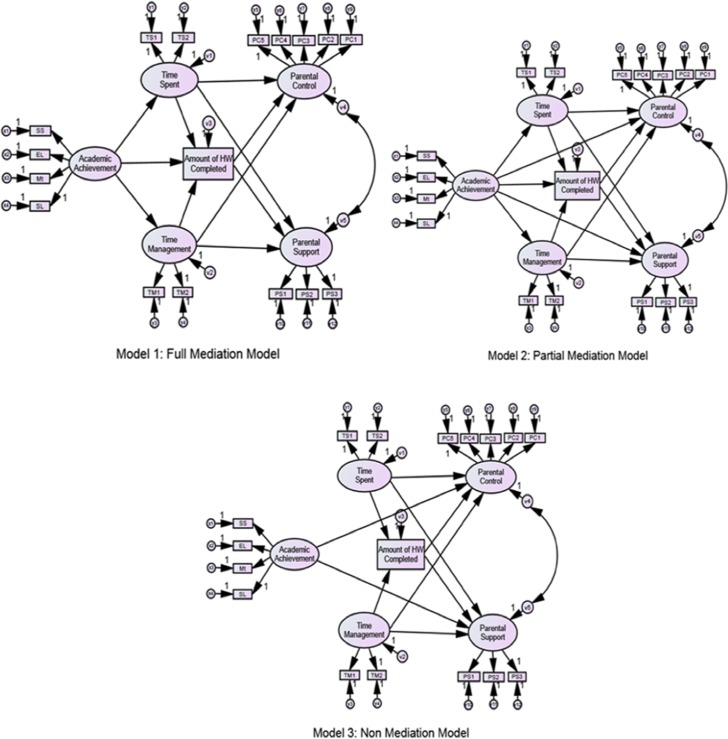
Structural equation model (SEM) of children’s academic functioning and homework parental involvement (full, partial, and non-mediation models). PC1,..., PC5 (measures of perceived Parental Control), PS1,..., PS3 (measures of perceived Parental Support), TS1 and TS2 (measures of Time Spent on HW Completion), TM1 and TM2 (measures of HW Time Management), HWC (measure of Amount of HW Completed), SL (measure of Spanish Language Achievement), Mt (measure of Mathematics Achievement), EL (measure of English Language Achievement), SS (measure of Social Sciences Achievement). V1 to V5 represent the variance explained. X1 to X5 and Y1 to Y12 are measurement errors.

To address the research questions of this study, data were analyzed in several stages. First, we calculated and analyzed descriptive statistics of the variables in the homework model. Second, following Núñez et al. (2015), three models were compared to examine to what extent the students’ homework behaviors mediated the association between students’ prior academic achievement and perceived parental involvement in homework: a full mediation model (M1), a partial mediation model (M2) [M1 plus a direct path from prior academic achievement to perceived parental involvement in homework (control and support)], and a non-mediation model (M3) [only the direct path from prior academic achievement to homework parental involvement] (see **Figure 2**). Information criteria-based model selection tools were used to compare the fit to the data of the three candidate models, and select the best (see Vallejo et al., 2014).

Third, multi-group analyses were conducted to check the invariance of the homework model chosen for the two subgroups of students at the middle and high school levels. Finally, the best-fit model was used to examine the three hypotheses of the study.

To account for the hierarchical structure of the data (i.e., students in classes), the homework model was fitted with Mplus 5.1 (Muthén et al., 1998–2007) using “type = complex” in the analysis command and “cluster = class” in the variable command. This procedure allowed computation of the standard errors and chi-square tests of model fit, taking into account clustering information and/or non-independence of observations, such as adjusting the standard errors of the regression coefficients. The MLR estimator in Mplus 5.1 (maximum likelihood robust) was selected, which is sensitive to non-normality and non-independence of observations.

A series of statistics and indices were used at different stages of data analysis. Akaike’s (1974) Akaike’s information criterion (AIC), Raftery’s (1993), Bayesian information criterion (BIC), and Browne and Cudeck’s (1993), Browne- and Cudeck’s criterion (BCC) were used to select the proper mediation model. Then, to assess the fit of the model chosen, in addition to chi-square (χ^2^) statistics and their associated probability (*p*) values, we used two absolute indices, the goodness-of-fit-index (*GFI*) and the adjusted goodness-of-fit-index (*AGFI*); a relative index, the Tucker Lewis Index (*TLI*) and the comparative fit index (*CFI*) (Bentler, 1990); and a close-fit parsimony-based index, the root mean square error of approximation (*RMSEA*), and their 90% confidence intervals (Hu and Bentler, 1999). According to these authors, a model fits well when: *GFI*, *AGFI*, and *TLI* > 0.90, CFI > 0.95, and *RMSEA* ≤ 0.05.

## Results

### Descriptive Data

**Table 1** shows descriptive statistics and the correlation matrix for the observed variables in the model. The variables are significantly inter-correlated. Because maximum likelihood (ML) can produce biases when variables fail to follow a normal distribution, we examined the distributions of all the variables (i.e., kurtosis and skewness). Taking the criterion of Finney and DiStefano (2006), for whom 2 and 7 are the maximum allowable values for skewness and kurtosis, respectively, all of the variables respected those criteria (see **Table 1**). Therefore, with normality conditions met, we fitted the model using MLR.

**Table 1:** Descriptive statistics of the variables in the structural equation homework model (*n* = 1250 middle and high school students).

	SL	PMT	EL	SS	TS1	TS2	TM1	TM2	HWC	PC1	PC2	PC3	PC4	PC5	PS1	PS2	PS3
SL	-																
PMT	0.75^∗∗^	-															
EL	0.75^∗∗^	0.68^∗∗^	-														
SS	0.77^∗∗^	0.66^∗∗^	0.75^∗∗^	-													
TS1	0.16^∗∗^	0.11^∗∗^	0.11^∗∗^	0.16^∗∗^	-												
TS2	0.33^∗∗^	0.25^∗∗^	0.31^∗∗^	0.34^∗∗^	0.54^∗∗^	-											
TM1	0.25^∗∗^	0.24^∗∗^	0.20^∗∗^	0.23^∗∗^	0.12^∗∗^	0.14^∗∗^	-										
TM2	0.26^∗∗^	0.22^∗∗^	0.21^∗∗^	0.25^∗∗^	0.16^∗∗^	0.20^∗∗^	0.56^∗∗^	-									
HWC	0.43^∗∗^	0.40^∗∗^	0.32^∗∗^	0.36^∗∗^	0.42^∗∗^	0.35^∗∗^	0.36^∗∗^	0.38^∗∗^	-								
PC1	-0.09^∗∗^	-0.05	-0.09^∗∗^	-0.09^∗∗^	0.13^∗∗^	0.08^∗∗^	0.04	0.08^∗∗^	0.17^∗∗^	-							
PC2	0.09^∗∗^	0.09^∗∗^	0.06^∗^	0.09^∗∗^	0.18^∗∗^	0.15^∗∗^	0.13^∗∗^	0.18^∗∗^	0.28^∗∗^	0.46^∗∗^	-						
PC3	-0.05	-0.01	-0.07^∗∗^	-0.01	0.22^∗∗^	0.13^∗∗^	0.11^∗∗^	0.17^∗∗^	0.28^∗∗^	0.45^∗∗^	0.47^∗∗^	-					
PC4	0.01	0.04	0.00	0.04	0.27^∗∗^	0.18^∗∗^	0.14^∗∗^	0.20^∗∗^	0.31^∗∗^	0.37^∗∗^	0.37^∗∗^	0.52^∗∗^	-				
PC5	-0.01	0.04	0.00	0.02	0.16^∗∗^	0.11^∗∗^	0.10^∗∗^	0.11^∗∗^	0.21^∗∗^	0.37^∗∗^	0.39^∗∗^	0.43^∗∗^	0.49^∗∗^	-			
PS1	0.03	0.01	-0.01	0.02	0.17^∗∗^	0.17^∗∗^	0.11^∗∗^	0.16^∗∗^	0.21^∗∗^	0.34^∗∗^	0.39^∗∗^	0.37^∗∗^	0.28^∗∗^	0.23^∗∗^	-		
PS2	0.11^∗∗^	0.10^∗∗^	0.08^∗∗^	0.10^∗∗^	0.15^∗∗^	0.18^∗∗^	0.07^∗^	0.13^∗∗^	0.23^∗∗^	0.30^∗∗^	0.33^∗∗^	0.32^∗∗^	0.26^∗∗^	0.23^∗∗^	0.52^∗∗^	-	
PS3	0.16^∗∗^	0.15^∗∗^	0.12^∗∗^	0.15^∗∗^	0.17^∗∗^	0.23^∗∗^	0.13^∗∗^	0.22^∗∗^	0.28^∗∗^	0.32^∗∗^	0.39^∗∗^	0.37^∗∗^	0.33^∗∗^	0.26^∗∗^	0.55^∗∗^	0.62^∗∗^	-
*M*	5.60	5.22	5.92	5.95	3.21	3.08	3.22	3.21	4.15	3.45	3.73	3.24	3.33	2.99	3.48	3.53	3.49
*SD*	2.18	2.20	2.33	2.31	1.15	1.30	1.12	1.07	0.97	1.32	1.14	1.43	1.47	1.44	1.36	1.39	1.34
*Skewness*	0.00	-0.04	-0.20	-0.19	-0.15	0.02	-0.30	-0.25	-1.24	-0.47	-0.67	-0.23	-0.30	0.03	-0.50	-0.54	-0.53
*Kurtosis*	-0.71	-0.68	-0.55	-0.66	-0.76	-1.10	-0.49	-0.51	1.01	-0.90	-0.29	-1.25	-1.27	-1.31	-0.95	-0.96	-0.86

*PC1,..., PC5 (measures of Perceived Parental Control), PS1,..., PS3 (measures of Perceived Parental Support), TS1 and TS2 (measures of Time Spent on HW Completion), TM1 and TM2 (measures of HW Time Management), HWC (measure of Amount of HW Completed), SL (measure of Spanish Language Achievement), PMT (measure of Prior Mathematics Achievement), EL (measure of English Language Achievement), SS (measure of Social Sciences Achievement). ^∗^*p* < 0.05; ^∗∗^*p* < 0.01*.

### Selecting the Best Model

The analyses of the comparison models showed that the fit of the non-mediation model was the worst of the three models (see **Table 2**). By comparison, the partial mediation model and the full mediation model showed a satisfactory fit, with the best fit of all provided by the partial mediation model [Δχ^2^(2) = 68.23, *p* < 0.001]. The likelihood ratio test procedure was favorable to the partial mediation model (M2). Also, to select the best fit model, the statistics provided by AIC, BIC, and BCC were used to determine which of the two models (full or partial mediation model) was more likely to accurately describe the relationships in the matrix data.

**Table 2:** Results of homework model comparison strategy.

	Models		
	Full mediation Model (M1)	Partial mediation Model (M2)	Non-mediation Model (M3)
NP	45	47	44
DF	108	106	109
χ^2^	548.48	480.25	779.31
χ^2^/DF	5.08	4.53	7.32
P	0.001	0.001	0.001
GFI	0.948	0.955	0.929
AGFI	0.926	0.936	0.901
TLI	0.938	0.946	0.904
CFI	0.951	0.958	0.923
RMSEA (90% CI)	0.057 (0.052–0.062)	0.053 (0.048–0.058)	0.071 (0.067–0.076)
AIC	638.48	574.25	885.31
BCC	329.32	265.15	576.13
BIC	59.63	5.66	301.34

*NP, number of parameters; DF, degrees of freedom; χ^*2*^, chi-square; GFI, goodness-of-fit-index; AGFI, adjusted goodness-of-fit-index; TLI, Tucker Lewis Index; CFI, comparative fit index; RMSEA, root mean square error of approximation; AIC, Akaike’s Information Criterion; BCC, Browne-Cudek’s Criterion; BIC, Bayesian Information Criterion*.

**Table 2** shows that the partial mediation model has a more valid BIC value than does the full mediation model. Similarly, efficient criteria (i.e., AIC) which tends to choose more complex models (Vallejo et al., 2014), as well as consistent criteria (i.e., BIC), which tends to choose simpler models, favored the selection of the Partial Mediation Model (M2). Based on the suggestions by Burnham and Anderson (2002), we selected M2 as the actual Kullback–Leibler best model for the population of possible samples.

### Grade Level Invariance Analysis

The hypothesis of the invariance of the Homework Partial Mediation Model in the two samples of students (7th -8th grade vs. 9th -10th grade) was analyzed with multi-group analyses. Specifically, we tested the similarity of the Homework Partial Mediation Model in both samples with regard to its five dimensions: measurement weights, structural weights, structural covariances, structural residuals, and measurement residuals.

Results showed that the hypothesized homework model is similar in both samples on four of the five criteria (see **Table 3**). Assuming that the unconstrained model is correct [χ^2^ = 577.614, df = 212, *p* < 0.001, χ^2^/df = 2.725, GFI = 0.948, AGFI = 0.924, CFI = 0.957, RMSEA = 0.037, 90% CI (0.034, 0.041), *p* = 1.000], when testing equality in measurement weights, in structural weights, in structural covariances, and in structural residuals no statistically significant differences were found. Finally, assuming the absence of differences in structural residuals, statistically significant differences were found in measurement residuals.

**Table 3:** Results of grade level invariance analysis.

	Δχ^2^	ΔDF	P	NFI	IFI	RFI	TLI
Measurement weights	27.199	14	0.018	0.003	0.003	-0.001	-0.002
Structural weights	15.074	10	0.129	0.002	0.002	-0.002	-0.002
Structural covariances	0.000	1	0.999	0.000	0.000	0.000	0.000
Structural residuals	6.424	5	0.267	0.001	0.001	-0.001	-0.001
Measurement residuals	79.683	17	0.000	0.009	0.009	0.004	0.004

*Δχ^*2*^, difference in χ^*2*^ values between models; ΔDF, difference in number of degrees of freedom between models; P, statistical significance; NFI, normed fit index; IFI, index of fit; RFI, relative fit index; TLI, Tucker Lewis Index*.

Therefore, the results show that the Homework Partial Mediation Model is invariant for the two groups of students in the first four dimensions (measurement weights, structural weights, structural covariances, and structural residuals), but not for the last one (measurement residuals). The analysis of structural weights and structural covariances was the main focus of the multi-group analysis, which indicates the appropriateness of using the total sample to analyze the homework model.

### Children’s Prior Academic Achievement and Perceived Parental Involvement in Homework

**FIGURE 3:**
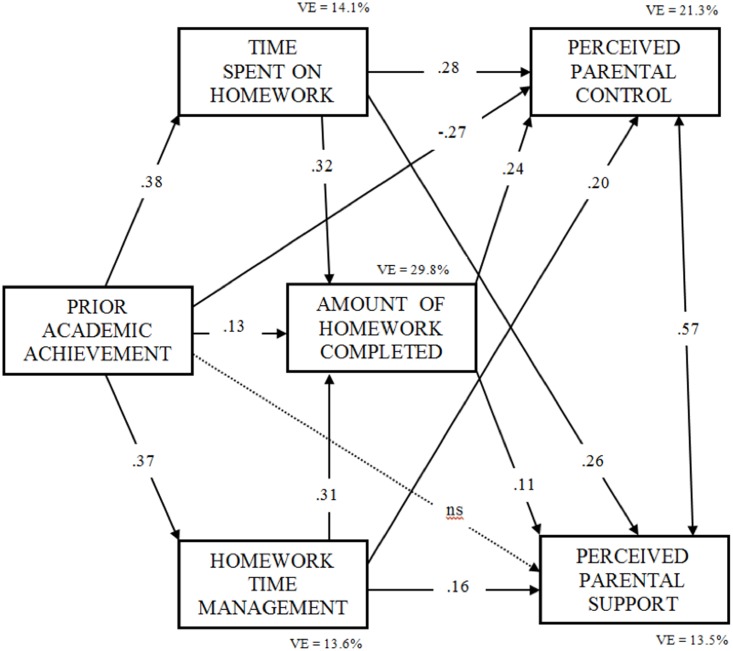
Standardized total effects in the Homework Partial Mediation Model (*N* = 1250). VE (Variance Explained). All coefficients are statistically significant at *p* < 0.001, except Homework Time Management on Perceived Parental Support (*p* < 0.01), and Prior Academic Achievement on Perceived Parental Support (*p* > 0.05, not statiscally significant).

Results for the Homework Partial Mediation Model adjustment are provided in **Table 4** and **Figure 3**. Overall, the analyses confirm the three hypotheses initially established for the study. First, as hypothesized, prior academic achievement was significantly associated with students’ homework behaviors. Statistically significant and positive associations were found between prior academic achievement and the time students spend on homework, the management of this time, and the amount of homework completed.

**Table 4:** Standardized and unstandardized regression weights, standard errors, *z*-values, and associated *p*-values for the Homework Partial Mediation Model.

			SRW	URW	SE	SRW/SE	*p*-value
Prior academic achievement	→	HW time spent	0.375	0.154	0.049	7.619	0.000
Prior academic achievement	→	HW time management	0.369	0.150	0.030	12.297	0.000
Prior academic achievement	→	Amount of HW completed	0.126	0.074	0.056	2.255	0.004
HW time spent	→	Amount of HW completed	0.317	0.452	0.069	4.592	0.000
HW time management	→	Amount of HW completed	0.310	0.446	0.037	8.453	0.000
HW time spent	→	Parental control	0.277	0.276	0.049	5.682	0.000
HW time spent	→	Parental support	0.255	0.293	0.048	5.344	0.000
HW time management	→	Parental control	0.196	0.197	0.051	3.859	0.000
HW time management	→	Parental support	0.160	0.186	0.045	3.584	0.000
Amount of HW completed	→	Parental control	0.243	0.170	0.045	5.384	0.000
Amount of HW completed	→	Parental support	0.105	0.085	0.044	2.383	0.017
Prior academic achievement	→	Parental control	-0.269	-0.110	0.043	-6.322	0.000
Prior academic achievement	→	Parental support	-0.037	-0.017	0.042	-0.884	0.377
Parental control	↔	Parental support	0.573	0.356	0.029	20.031	0.000

*Data of measurement model (relation between the observed variables and the corresponding latent variables) are not included. SRW, standardized regression weights; URW, unstandardized regression weights; SE, standard error*.

Second, children’s homework variables and perceived parental homework involvement were significantly and positively related: time spent on homework with perceived parental control and with perceived parental support; time homework management with perceived homework parental control and with perceived homework parental support; and, finally, amount of homework completed with perceived homework parental control and with perceived homework parental support. Third, the direct association between prior academic achievement and perceived parental involvement in homework was significant and negative for perceived parental control, but contrary to our hypothesis not statistically significant for perceived parental support. It is interesting to note, however, that the indirect association between prior performance and perceived homework parental involvement (through time spent on homework, homework time management, and amount of homework completed) was positive and significant for both types of perceived homework parental involvement: support and control. Four, both dimensions of perceived parental involvement in homework were positive and strongly related (*r* = 0.573, *d* = 1.40).

Additionally, data indicate that both dimensions of perceived parental homework involvement also were moderately predicted by children’s achievement levels and students’ homework behaviors (see **Figure 3**): 21.3% (perceived homework parental control) and 14.1% (perceived homework parental support).

## Discussion

Pomerantz et al. (2007, p. 399) suggested that research is needed on how children’s characteristics influence their interactions with and the involvement of parents on school work. In their words: “[the] consideration of the match between children’s characteristics and the manner in which parents become involved is a crucial endeavor.” Their call identified an important research agenda that has not been adequately addressed. Parental involvement does not “produce” student achievement. Rather the parents’ attitudes and actions must flow to and through students, who must interpret and respond to the involvement activities with their own attitudes and actions.

This study responds to that call by focusing on whether student characteristics affect their views of parental involvement. Tests of the data favored SEM analysis of a partial mediation model to explore connections of students’ prior levels of achievement, homework behaviors, and perceived parental involvement in homework. Two major topics emerged that are important to discuss.

### Prior Academic Achievement and Students’ Homework Behaviors Predict Perceived Parental Involvement in Homework

This study explored the connections of middle and high school students’ prior levels of achievement and reported homework behaviors with students’ perceptions of the nature of their own parents’ involvement in homework. Findings indicated that level of achievement was related to students’ perceptions of how their parents behaved about homework. Specifically, the data showed that the higher the students’ prior achievement, the more time they spent on their homework, the more homework was completed, and the better their homework time management. Further, the more time spent and the more homework completed, the stronger students’ reports of their parents’ involvement in terms of both control and support of homework.

These findings are aligned with other studies that examined the relationship of these variables in the opposite, more common direction. For example, Núñez et al. (2015), found that the more students’ reported their parents’ involvement, the more time they spent doing homework, the better their time management, and the higher their academic achievement.

By examining different assumptions about the direction of influence of children’s characteristics and parents’ engagement in homework, we can see that, however viewed, students with higher prior achievement tend to spend more time on homework, manage it better, and do more homework. With achievement level taken into account, students who take time to do their homework, perceive and report that their parents continue to offer controlling and supportive messages about the homework process.

This study reinforced prior findings that when secondary school students’ academic performance is poor, they tend to spend less time doing homework, manage their time less effectively, and complete less homework. This study extends prior results by showing that low-achieving students perceived and reported that their parents proffered more controlling messages about homework. Pomerantz et al. (2005b) claimed that children with a history of poor academic performance may be particularly sensitive to the quantity and quality of parental involvement. Low-achieving students, even at the secondary level, may need extra attention from parents to keep them invested in the homework process. If parental involvement is more controlling for these children as suggested in this study and by Núñez et al. (2015), the low-achieving students may progressively disengage from their homework and school tasks. Longitudinal data are needed to examine if parents’ extra pressure helps low achievers improve their achievement scores and stay in school compared to low achievers whose parents ignore the homework process.

### The Direct Relationships of Students’ Levels of Prior Academic Performance with Parental Involvement in Homework Differ for Perceived Parental Controlling and Supportive Behaviors

As in prior investigations, this study found a direct negative relationship between children’s academic performance and students’ perceptions of parental involvement in homework, particularly parents’ controlling behaviors. With other variables statistically controlled, students with lower achievement reported that their parents conducted more monitoring and controlling behaviors about homework. There was no significant relationship of student achievement with perceived parental support of homework.

Some researchers explain this pattern of results as reflecting parents’ recognition that low-achieving children need more direction and control than do more successful students, who take more personal responsibility for completing their homework (Pomerantz and Eaton, 2001; Grolnick et al., 2002; Pomerantz et al., 2005a; Epstein and van Voorhis, 2012). Others explain that some parents lack confidence and competence to guide their children in other ways than by controlling (Hoover-Dempsey and Sandler, 1997). Still, others suggest that parents will be more controlling if they and the children have negative attitudes toward homework or behavior problems while doing homework (Fuligni et al., 2002; Pomerantz et al., 2005a), or if parents feel less competent to help children work independently on homework (Pomerantz and Eaton, 2001), or if the child and parents area frustrated by persistent low school achievement (Pomerantz et al., 2005a). As Pomerantz et al. (2007, p. 383) note “when parents’ involvement is controlling, children do not have the experience of solving challenges on their own,” and, “when parents are controlling, they may deprive children of feeling that they are autonomous, effective agents.”

A pattern of over-control by parents may not help students who are struggling to improve their achievement. Several studies reported a connection of high control of homework by parents and children low academic achievement (Cooper et al., 2000; Dumont et al., 2012; Karbach et al., 2013; Núñez et al., 2015). These students may be particularly sensitive to parents’ pressure about homework and may not understand the parents’ intent to motivate them to do their work. The findings from this and other research suggest the existence of a *vicious circle* in the relationship between children’s prior academic achievement, perceived parental involvement in control of homework, and children’s later achievement. That is, unless parental involvement in homework is carefully balanced with caring control and support messages, low-achieving students may avoid homework and disengage from school, especially in the secondary grades. To break the cycle, this study suggests, an optimal combination of control and support messages is needed to encourage middle and high school students to spend time on, manage, and complete their homework.

## Conclusion

A substantial amount of research has analyzed the association of various student homework behaviors (e.g., time spent on homework, time management, amount of homework done, procrastination, emotions, goals and motivations for doing homework, attitudes) with students’ academic achievement. Literature also is replete with studies of how parental involvement in homework affects students’ academic achievement. However, few studies *flipped the coin* to examine how students’ prior achievement levels affect their homework behaviors and how children’s academic functioning affects parents’ control or support of homework.

This study examined the little known associations of secondary students’ achievement levels with other important elements of the homework process—students’ behaviors and parents’ involvement. The findings indicated that children’s academic functioning was associated with their perceptions of parental involvement in the homework process. The study reveals the recursive nature of these important components of the homework process: children’s achievement level affects perceived parental involvement in homework, and, over time, parental involvement in homework affects students’ later performance.

This study supports and extends the results of past research to support an interactive model of socialization (Collins et al., 2000; Grusec, 2002). The behaviors of parents and children are modeled progressively as their interactions proceed and progress, and as results accumulate to shape the trajectory of student learning.

### Limitations

The presumed reciprocal relationships of parent involvement and student achievement are provocative, but they must be examined in future studies. This study’s data were cross-sectional with one-time measures of the independent and dependent variables. This prohibits claims of causality. Future studies using the required longitudinal data and/or experimental designs that guide specific parental behaviors and messages could test the assumption of recursive relationships of parental involvement and student achievement, which may affect each other, over time.

Another limitation of this study is that all measures were reported by the students. This helped us learn what students with different levels of achievement say about their homework time and products, and how they view the involvement of their parents. Although important, one set of reporters is not sufficient for fully understanding the roles and relationships of students and parents that affect the homework process. Behavior-based measures from students (such as a homework diary) and data from parents of their involvement in homework are needed, along with the children’s views, to study whether multiple reporters explain their behaviors in the same way. Multiple measures from multiple reporters would confirm or challenge the accuracy of reports of students’ homework behaviors of time spent and homework completed, and build a more robust understanding of the complex and continuous influences on student achievement.

### Applications

Although research on all aspects of the homework process must continue to improve, the results of this and prior studies (e.g., Grolnick and Slowiaczek, 1994; Epstein, 1995; Cooper and Valentine, 2001; Hill and Taylor, 2004; Pomerantz et al., 2005b; Epstein, 2007), have clear and useful educational applications. Numerous studies confirm that, over time, parental involvement with students on homework is associated with higher student achievement. Positive practices of parental involvement may promote students’ cognitive, linguistic, and mathematical skills, metacognitive skills, and strategies for a self-regulated learning, as well as positive attitudes toward school and motivation to learn. However, as Darling and Steinberg (1993) alerted, the effect of these practices is largely determined by the style in which the practices are carried out. And, results also depend on the quality of the design and clarity of parental involvement activities on homework with specific learning goals for students (Epstein and van Voorhis, 2012). The present study and previous research suggest that parental involvement in homework should be more strongly characterized by autonomy support, process focus, positive affect, and positive beliefs in students’ abilities than by too much control, negative affect, and negative beliefs about homework. School administrators, school psychologists, and teachers should offer workshops for secondary school parents on core aspects of their involvement in homework (e.g., how to support students’ independent thinking and completion of assignments; how to prevent and cope with children’s emotional distress about homework; and how to maintain student motivation but reduce undue parental pressure, particularly on students who are struggling in school).

The results of this and other studies suggest that, with teachers’ guidance and materials, more parents could help their students (a) strive to be more independent in their study (Núñez et al., 2013a); (b) understand that their effort (not innate ability) will help them complete their assignments; (c) focus on the positive aspects of school, homework, and learning rather than on negative attitudes (Cunha et al., 2015); and (d) face homework with self-confidence not just to avoid failure but to complete tasks, solve problems, and meet success. The results of this study deepen our understanding about the potential for parents’ positive interactions with their teens on homework at the secondary school level.

## Ethics Statement

This study was carried out in accordance with the recommendations of the ethics committee of the University of Oviedo. All subjects gave written informed consent in accordance with the Declaration of Helsinki.

## Author Contributions

NS was responsible for the data collection, and for the literature search with JN. GV was responsible for data analysis, and JE for the data interpretation. JN, JE, PR, and AV made important intellectual contribution in research design and manuscript revision. All authors were involved in the writing process of this manuscript.

## Conflict of Interest Statement

The authors declare that the research was conducted in the absence of any commercial or financial relationships that could be construed as a potential conflict of interest.
